# PSIP1 Orchestrates Immune Escape in Osteosarcoma: Insights From Single‐Cell Analysis and Implications for Immunotherapy Resistance

**DOI:** 10.1155/ijog/2344901

**Published:** 2026-05-22

**Authors:** Simin Liu, Hong Yan, Wei Ku, Guo Liu

**Affiliations:** ^1^ Department of Spinal Surgery, Xianning Central Hospital, The First Affiliated Hospital of Hubei University of Science and Technology, Xianning, Hubei, China

## Abstract

**Background:**

Osteosarcoma (OS) is characterized by a highly heterogeneous tumor microenvironment (TME) that frequently limits the efficacy of immunotherapy. Identifying molecular drivers of immune escape (IE) is essential for improving therapeutic outcomes.

**Methods:**

We integrated single‐cell RNA sequencing (scRNA‐seq) and bulk transcriptomic data to dissect the OS immune landscape. Utilizing high‐dimensional weighted gene coexpression network analysis (hdWGCNA), we identified gene modules specifically associated with IE.

**Results:**

Within the IE‐related module, PSIP1 emerged as the most significant prognostic hub gene. ScRNA‐seq analysis localized PSIP1 primarily to malignant cell populations. High PSIP1 expression consistently correlated with abbreviated overall survival across multiple independent cohorts. Functional enrichment and GSEA revealed that PSIP1 is intrinsically linked to the suppression of critical immune effectors, including B‐cell signaling, lymphocyte chemotaxis, and NK cell–mediated cytotoxicity. Crucially, PSIP1 exhibited robust predictive performance for immunotherapy response; elevated PSIP1 levels were associated with diminished activity of multiple established immune‐response signatures and poor survival in patients undergoing checkpoint blockade.

**Conclusion:**

Our study positions PSIP1 as a novel orchestrator of immune evasion and a promising predictive biomarker for immunotherapy resistance in OS, offering a potential target to overcome therapeutic recalcitrance.

## 1. Introduction

Osteosarcoma (OS) is the most common primary bone malignancy in children and adolescents, characterized by high metastatic potential and heterogeneous clinical outcomes [1]. Despite advances in multimodal therapy [2, 3], survival rates for patients with recurrent or metastatic disease remain poor, underscoring the need for novel therapeutic strategies [4–7]. Immunotherapy has emerged as a promising approach for various cancers; however, its efficacy in OS is limited, partly due to the complex and immunosuppressive tumor microenvironment (TME) [8]. A deeper understanding of the cellular composition of the TME [9, 10] and the molecular drivers of immune escape (IE) is essential to improve patient stratification and therapeutic response.

The TME of OS comprises diverse cell types, including malignant cells, immune cells, and stromal components, which interact to foster tumor progression and immune evasion [8, 11]. Recent advances in single‐cell RNA sequencing (scRNA‐seq) have enabled detailed characterization of this heterogeneity [12, 13]. Concurrently, bioinformatics approaches such as WGCNA facilitate the identification of gene modules associated with specific biological traits, including IE [14].

PSIP1, also known as LEDGF/p75, is a critical nuclear protein that functions primarily as a transcriptional coactivator and a molecular tether, playing an indispensable role in integrating retroviral DNA such as HIV‐1 into the host genome by binding tightly to the viral integrase and directing it to active transcription units [15, 16]. Its dysfunction is implicated in various diseases, including autoimmune disorders such as psoriasis and certain leukemias, in which chromosomal translocations can create oncogenic fusion proteins [17]. Although its role has been studied in other cancers, its function in OS, particularly within the context of the immune TME and immunotherapy response, remains largely unexplored.

In this study, we used scRNA‐seq and bulk transcriptomic data to deconvolve the OS TME and identify IE‐related gene networks. We focused on PSIP1 as a key hub gene derived from these analyses and systematically evaluated its prognostic significance, biological functions, and predictive value for immunotherapy response. Our work is aimed at establishing PSIP1 as a biomarker with potential clinical utility in guiding prognosis and immunotherapeutic strategies for OS patients.

## 2. Materials and Methods

### 2.1. Data Acquisition and Preprocessing

The study integrated multiple transcriptomic dimensions to investigate OS. scRNA‐seq data (GSE152048), comprising 123,587 cells, were retrieved from the Gene Expression Omnibus (GEO) [11]. For clinical validation, bulk RNA‐seq datasets and corresponding pathological information were obtained from the Therapeutically Applicable Research to Generate Effective Treatments (TARGET‐OS) database and the GEO database (GSE21257) [18]. All bulk data were normalized and log2‐transformed to ensure interbatch comparability.

### 2.2. Single‐Cell Landscape and Malignant Cell Identification

The scRNA‐seq analysis was conducted using the R package Seurat (v4.0) [19]. For scRNA‐seq data quality control, we retained cells that met the following criteria: < 20% of counts mapped to mitochondrial genes and < 5% mapped to ribosomal genes, to remove low‐quality cells. Following data normalization and principal component analysis (PCA), cell clustering was visualized using t‐distributed Stochastic Neighbor Embedding (t‐SNE). To distinguish malignant cells from normal stromal components, the inferCNV package was employed to calculate copy number variation (CNV) profiles, using lymphoid and myeloid cells as reference baselines. Cell type–specific markers were identified through the FindAllMarkers function to annotate the diverse TME populations.

### 2.3. High‐Dimensional WGCNA and Hub Gene Selection

To capture the coordinated gene expression patterns associated with immune evasion, we performed high‐dimensional weighted gene coexpression network analysis (hdWGCNA) on the scRNA‐seq dataset [20]. Unlike traditional WGCNA, hdWGCNA constructs coexpression networks specifically for single‐cell data by aggregating cells into “metacells.” A scale‐free topology model was established with a calculated soft‐power threshold. We then correlated module eigengenes with a curated list of IE‐related genes [21]. The module with the highest positive correlation was prioritized, and univariate Cox regression analysis was performed on its members to identify PSIP1 as the top‐ranked prognostic hub gene.

### 2.4. Functional Enrichment and Immunotherapy Response Prediction

The biological implications of PSIP1 were explored using Gene Set Enrichment Analysis (GSEA) against the Gene Ontology (GO) database and pathway annotations via the Metascape platform [22]. To assess the clinical utility of PSIP1 in immunotherapy, we calculated its correlation with nine validated immunotherapy response signatures (e.g., CYT, IFN*γ*IS, and ICBnetIS) [23–25]. Furthermore, the predictive accuracy of PSIP1 was rigorously validated across four independent immunotherapy cohorts (Ascierto, Kim, Cho, and Nathanson) using receiver operating characteristic (ROC) curves and Kaplan–Meier survival analysis to assess its potential to predict treatment resistance.

## 3. Results

### 3.1. Decoding the Cellular Heterogeneity of the OS TME

**Figure 1:**
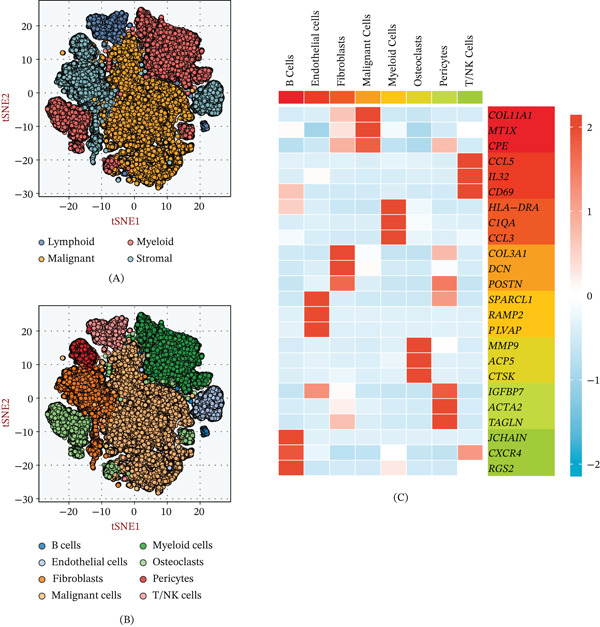
Single‐cell transcriptomic landscape of the osteosarcoma tumor microenvironment (TME). (A) t‐SNE visualization identifying four primary cell lineages within the osteosarcoma TME, including lymphoid, malignant, myeloid, and stromal compartments. (B) High‐resolution t‐SNE clustering partitions the TME into eight distinct cell subpopulations. (C) Heatmap highlighting the expression profiles of canonical marker genes across the identified cell types, confirming the fidelity of cluster annotations.

To resolve the intricate landscape of the OS microenvironment, we executed a comprehensive single‐cell transcriptomic analysis. Dimensionality reduction via t‐SNE successfully partitioned the cellular constituents into four foundational lineages: lymphoid, malignant, myeloid, and stromal cells (Figure 1A). A higher resolution clustering strategy further revealed eight distinct subpopulations, notably featuring malignant cells, T/NK cells, fibroblasts, and osteoclasts (Figure 1B). The fidelity of this cell‐type annotation was corroborated by a heatmap of lineage‐specific marker genes, which demonstrated clear transcriptomic separation between these clusters (Figure 1C).

### 3.2. Identification of IE‐Centric Gene Networks via hdWGCNA

**Figure 2:**
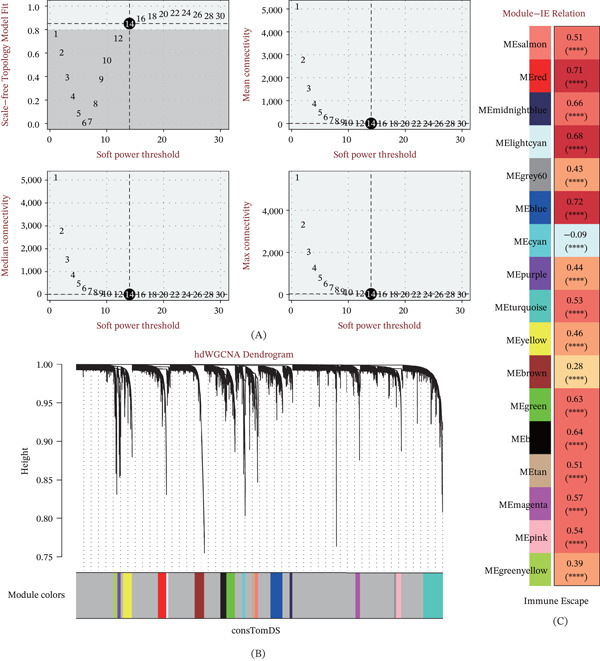
Identification of immune escape (IE)–associated gene modules via hdWGCNA. (A) Analysis of network topology for various soft‐thresholding powers, ensuring the construction of a scale‐free coexpression network. (B) Hierarchical clustering dendrogram illustrating the distribution of 17 distinct gene coexpression modules. (C) Correlation matrix between module eigengenes and the IE signature; the blue module demonstrates the strongest positive correlation.

Recognizing that immune evasion is a coordinated programmatic shift, we leveraged hdWGCNA to identify coexpression modules synchronized with the IE process. By establishing a scale‐free topology with a soft‐power threshold of 14 (Figure 2A), we segmented the high‐dimensional data into 17 coexpression modules (Figure 2B). Among these, the “blue module” exhibited the most pronounced positive association with the IE signature (*r* = 0.72, Figure 2C), suggesting that the genes within this cluster are primary drivers of the immunosuppressive architecture in OS.

### 3.3. PSIP1 as a Crucial Prognostic Determinant in OS

**Figure 3:**
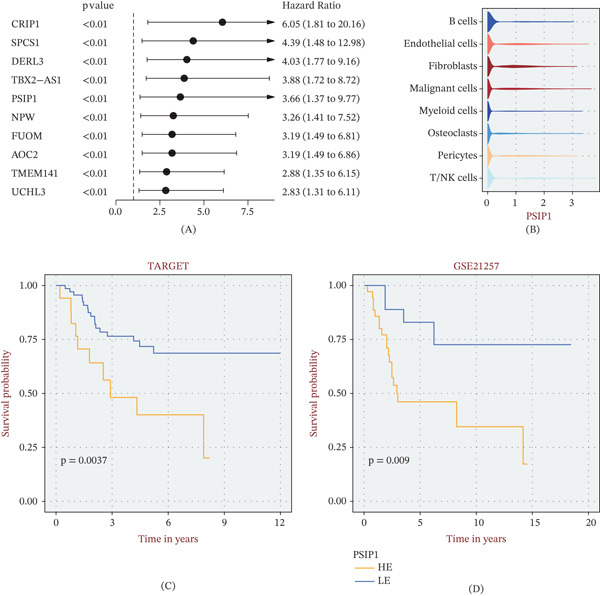
Evaluation of the prognostic significance and cellular localization of PSIP1. (A) Forest plot of univariate Cox regression analysis showing the Top 10 prognostic genes within the blue module, with PSIP1 exhibiting the highest hazard ratio (HR). (B) Violin plot depicting the expression levels of PSIP1 across diverse TME cell types, showing predominant enrichment in malignant cells. (C, D) Kaplan–Meier curves comparing overall survival between PSIP1‐high and PSIP1‐low groups in (C) the TARGET cohort and (D) the GSE21257 validation cohort.

We next sought to pinpoint the most clinically relevant drivers within the IE‐associated blue module. Univariate Cox regression identified 10 candidate genes with significant prognostic impact, with PSIP1 emerging as the strongest risk factor, owing to its lower hazard ratio (Figure 3A). Interestingly, single‐cell profiling revealed that PSIP1 expression was heterogeneous, with a predominant concentration in the malignant cell compartment (Figure 3B). This malignant‐specific upregulation translated into poor clinical outcomes: Survival analyses in both the TARGET (Figure 3C) and GSE21257 (Figure 3D) cohorts confirmed that elevated PSIP1 levels were associated with significantly shorter overall survival.

### 3.4. Functional Landscape and Immuno‐Inhibitory Role of PSIP1

**Figure 4:**
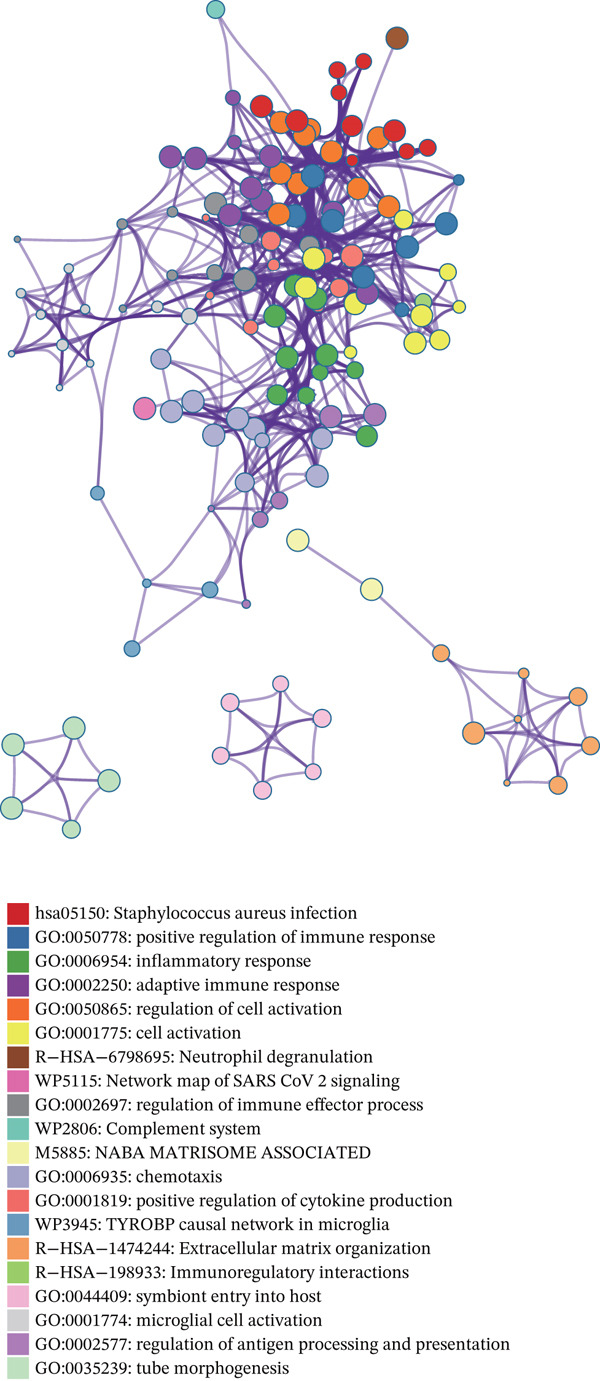
Biological pathway annotation and functional characterization of PSIP1. Metascape‐based enrichment analysis reveals the involvement of PSIP1 in critical pathways, including inflammatory responses, adaptive immunity, and neutrophil‐mediated processes.

**Figure 5:**
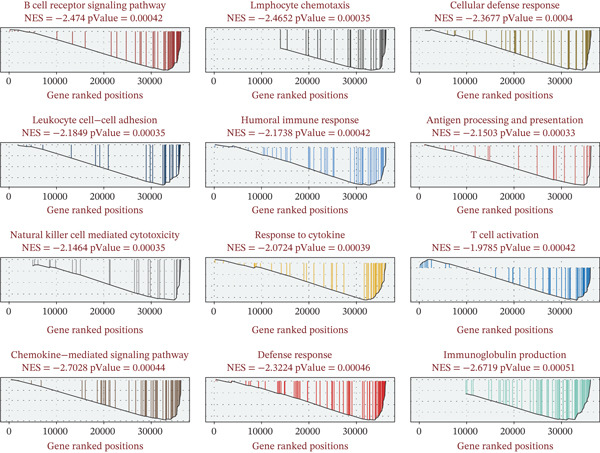
Gene Set Enrichment Analysis (GSEA) of PSIP1‐associated immune functions. Mountain plots showing 12 significantly enriched GO terms; high PSIP1 expression is characteristically associated with the suppression of B‐cell signaling, lymphocyte chemotaxis, and NK cell–mediated cytotoxicity.

To uncover the mechanism by which PSIP1 influences the TME, we performed pathway annotation via Metascape. PSIP1 was heavily involved in inflammatory modulation and adaptive immune responses (Figure 4). Deepening this inquiry through GSEA, we observed that high PSIP1 expression was inversely correlated with several immune‐activating hallmarks. Specifically, 12 GO terms were significantly suppressed, including crucial processes such as B‐cell receptor signaling, lymphocyte chemotaxis, and NK cell–mediated cytotoxicity (Figure 5). These findings imply that PSIP1 may act as a molecular brake on the host’s anti‐tumor immunity.

### 3.5. PSIP1 Predicts Therapeutic Resistance to Immunotherapy

**Figure 6:**
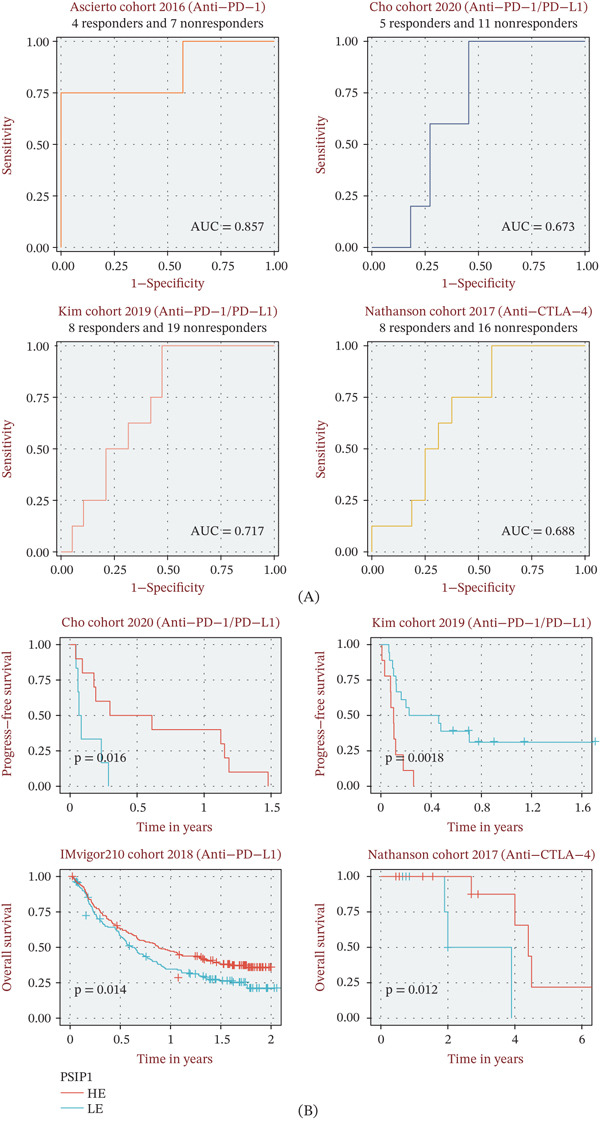
Predictive performance of PSIP1 in forecasting immunotherapy response. (A) receiver operating characteristic (ROC) curves evaluating the sensitivity and specificity of PSIP1 in predicting clinical response across four independent immunotherapy cohorts. (B) Kaplan–Meier survival analysis demonstrating the association between high PSIP1 expression and inferior clinical outcomes following immunotherapy.

**Figure 7:**
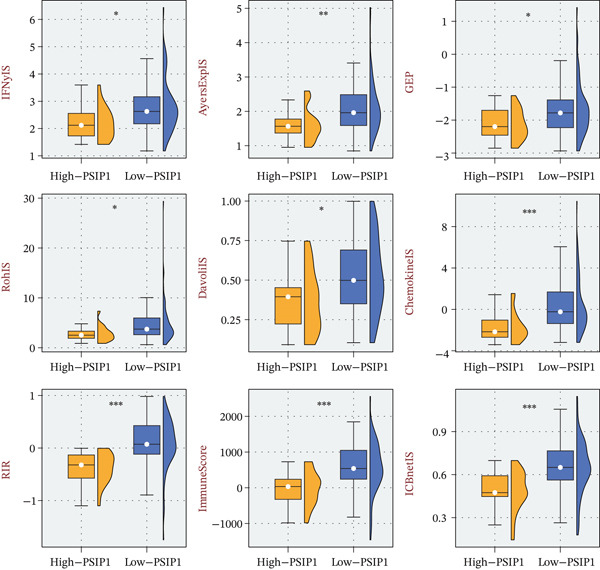
Impact of PSIP1 on established immunotherapy determinants. Box plots illustrating the differential expression of nine validated immunotherapy response signatures between PSIP1‐high and PSIP1‐low groups, showing a systematic downregulation in PSIP1‐high tumors.

Given its role in immune suppression, we evaluated whether PSIP1 could serve as a predictive gauge for immunotherapy efficacy. In four independent clinical trials (Ascierto, Kim, Cho, and Nathanson), PSIP1 demonstrated impressive discriminatory power, as evidenced by ROC analysis (Figure 6A). Patients categorized into the high‐PSIP1 group consistently exhibited inferior survival postimmunotherapy (Figure 6B). Furthermore, we observed a systemic depletion of multiple immunotherapy‐favoring signatures in tumors with high PSIP1 expression (Figure 7), solidifying its role as a robust biomarker for treatment resistance.

## 4. Discussion

In this study, we identified PSIP1 as a central gene associated with IE and poor prognosis in OS. By integrating scRNA‐seq data with network‐based bioinformatics, we demonstrated that PSIP1 is highly expressed in malignant cells and correlates significantly with adverse patient outcomes in independent cohorts. These findings position PSIP1 as a robust prognostic biomarker and shed light on its functional role in modulating the OS immune landscape.

Our hdWGCNA analysis revealed a specific gene module strongly linked to IE, with PSIP1 exhibiting the highest hazard ratio among module genes. This suggests that PSIP1 may play a driver role in immune evasion mechanisms. Pathway enrichment analysis further connected PSIP1 to key immune and inflammatory processes, including adaptive immune response, neutrophil degranulation, and cytokine regulation. These pathways are often dysregulated in immunosuppressive TMEs, supporting the notion that PSIP1 contributes to an immune‐hostile niche.

The GSEA results provided functional evidence that high PSIP1 expression is associated with the suppression of multiple immune‐activating pathways, including B‐cell receptor signaling, lymphocyte chemotaxis, and NK cell–mediated cytotoxicity [26, 27]. This broad dampening of immune effector functions aligns with the observed correlation between high PSIP1 levels and reduced activity of established immunotherapy response signatures. The downregulation of these immunotherapy response signatures in PSIP1‐high tumors directly contributes to multiple key immune evasion mechanisms: Reduced cytotoxicity of T and NK cells impairs the direct elimination of malignant cells; impaired lymphocyte chemotaxis leads to T‐cell exclusion from the TME; and suppressed B‐cell receptor signaling reduces the antitumor humoral immune response. Together, these effects create a profoundly immunosuppressive TME that drives resistance to ICB therapy. Importantly, PSIP1 demonstrated consistent predictive value across several immunotherapy cohorts, in which high expression was associated with poorer survival and reduced responsiveness to immune checkpoint inhibition. This suggests that PSIP1 could serve as a noninvasive biomarker to identify OS patients who are less likely to benefit from immunotherapy, thereby aiding treatment selection.

From a clinical perspective, PSIP1 holds significant utility for patient stratification in OS. First, PSIP1 can be used as a prognostic biomarker to identify high‐risk patients with poor survival outcomes at diagnosis, enabling more intensive surveillance and adjuvant treatment for this population. Second, PSIP1 can serve as a predictive biomarker to stratify patients for immunotherapy: Patients with low PSIP1 expression are more likely to benefit from ICB therapy, whereas patients with high PSIP1 expression may require alternative therapeutic strategies, such as combination therapy targeting PSIP1‐mediated immune suppression, to improve treatment response. Finally, PSIP1 can be used as a patient selection marker for future clinical trials of novel immunotherapies in OS to enrich the study population with patients who are most likely to respond to the experimental treatment.

To contextualize the novelty of our findings, we compared PSIP1 with previously reported immune‐related biomarkers in OS. The most widely studied biomarker for OS immunotherapy is PD‐L1; however, multiple studies have shown that PD‐L1 expression in OS is heterogeneous, and its predictive value for ICB response is inconsistent across cohorts. Other biomarkers, such as tumor‐infiltrating lymphocyte (TIL) density and chemokine signatures, are limited by the need for complex multigene testing or subjective pathological evaluation. In contrast, PSIP1 is a single‐gene biomarker that can be easily detected via routine quantitative PCR or immunohistochemistry, with robust prognostic value across two independent OS cohorts, and consistent predictive value for immunotherapy response across four independent ICB‐treated cohorts. These advantages make PSIP1 a more clinically feasible biomarker for OS than existing markers.

We also acknowledge the pharmacological challenges of targeting PSIP1, a nuclear transcriptional coactivator, with conventional small‐molecule inhibitors. However, several feasible therapeutic strategies can be explored to translate our findings into clinical applications. First, proteolysis‐targeting chimeras (PROTACs) have emerged as a powerful tool to degrade nuclear proteins that are considered “undruggable” with conventional inhibitors, and preclinical studies have successfully developed PROTACs targeting other transcriptional coactivators, providing a feasible approach to target PSIP1. Second, we can target the protein–protein interaction between PSIP1 and its transcriptional partners, which is required for its immune‐suppressive function. Third, we can target the downstream immune‐suppressive pathways regulated by PSIP1, such as the inhibition of lymphocyte chemotaxis and NK cell cytotoxicity, to reverse the immune‐suppressive TME in PSIP1‐high OS.

Our study has several limitations. The analyses were performed retrospectively using publicly available datasets, and further prospective validation in clinical cohorts is warranted. Additionally, although bioinformatics analyses strongly suggest PSIP1′s role in immune suppression, experimental studies using in vitro and in vivo models are needed to elucidate the precise molecular mechanisms by which PSIP1 regulates IE. The functional interaction between PSIP1 and specific immune cell populations within the TME also warrants further investigation. Another limitation of our study is that scRNA‐seq analysis does not capture the tumor′s spatial architecture. The spatial proximity and direct physical interaction between PSIP1‐expressing malignant cells and suppressed immune effector cells (such as NK cells and T cells) in the OS TME remain to be validated via spatial transcriptomics or multiplex immunofluorescence in future studies.

Despite these limitations, our findings provide a comprehensive bioinformatics framework linking PSIP1 to immune dysfunction and therapeutic resistance in OS. Future work should focus on validating PSIP1 as a clinical biomarker and exploring its potential as a therapeutic target to reverse immune evasion and enhance the efficacy of immunotherapy in OS patients.

## Author Contributions

S.L. and H.Y. contributed equally to this study. S.L. and H.Y. were responsible for data collection and analysis. W.K. drafted the manuscript, whereas G.L. conceptualized and oversaw the study. All authors contributed to the review of the final manuscript.

## Funding

No funding was received for this manuscript.

## Disclosure

All authors approved the final manuscript.

## Consent

The authors have nothing to report.

## Conflicts of Interest

The authors declare no conflicts of interest.

## Data Availability

All analytical data supporting the findings of this manuscript are available from the corresponding authors upon reasonable request.
